# At-Home Immersive Virtual Reality Exergames to Reduce Cardiometabolic Risk Among Office Workers: Protocol for a Randomized Controlled Trial

**DOI:** 10.2196/64560

**Published:** 2025-01-20

**Authors:** Jing Zhao, Akitomo Yasunaga, Andrew T Kaczynski, Hyuntae Park, Yufeng Luo, Jiuling Li, Ai Shibata, Kaori Ishii, Shohei Yano, Koichiro Oka, Mohammad Javad Koohsari

**Affiliations:** 1 School of Architecture and Urban Planning Guangzhou University Guangzhou China; 2 Faculty of Health Sciences Aomori University of Health and Welfare Aomori Japan; 3 Department of Health Promotion, Education, and Behavior Prevention Research Center, Arnold School of Public Health University of South Carolina Columbia, SC United States; 4 Department of Health Sciences Graduate School Dong-A University Busan Republic of Korea; 5 School of Advanced Science and Technology Japan Advanced Institute of Science and Technology Nomi Japan; 6 Institute of Health and Sport Sciences University of Tsukuba Tsukuba Japan; 7 Faculty of Sport Sciences Waseda University Saitama Japan; 8 Institute for Sport Sciences Waseda University Saitama Japan; 9 Human Care Research Team Tokyo Metropolitan Institute for Geriatrics and Gerontology Tokyo Japan; 10 School of Exercise and Nutrition Sciences Faculty of Health Deakin University Geelong Australia

**Keywords:** metabolic syndrome, noncommunicable diseases, active video game, interactive virtual reality environment, physical activity, workplace health, at-home intervention

## Abstract

**Background:**

The worldwide rise in the prevalence of noncommunicable diseases has increased the recognition of the need to identify modifiable risk factors for preventing and managing these diseases. The office worker, as a representative group of physically inactive workers, is exposed to risk factors for metabolic syndrome, which is a primary driver of noncommunicable diseases. The use of virtual reality (VR) exergames may offer a potential solution to the problem of increasing noncommunicable disease prevalence, as it can help individuals increase their physical activity levels while providing a more immersive experience.

**Objective:**

This exploratory study aims to examine the interventional efficacy of at-home immersive VR exergames on metabolic syndrome biomarkers among office workers. Additionally, it seeks to investigate the impacts of at-home immersive VR exergames on the active and sedentary behaviors of office workers.

**Methods:**

A 3-arm, single-blinded pilot randomized controlled trial will be conducted to examine the therapeutic effects of at-home immersive VR exergames. A total of 120 Chinese office workers, engaging in less than 150 minutes per week of moderate to vigorous intensity physical activity, will be recruited via a convenience sampling method. The participants, who will be tested over a 12-week period, will be randomly assigned to one of three groups: (1) the VR exergame intervention group, (2) the regular physical activity control group, and (3) the nonexercise control group. Throughout the 12-week trial, three categories of variables will be collected across the three groups: clinical risk factors associated with metabolic syndrome, active and sedentary behaviors, and demographics. To analyze variance among the groups, a mixed linear model will be applied to assess the efficacy of each group. Differences in metabolic syndrome clinical risk factors among all groups will be used to evaluate the effects of at-home immersive VR exergames. Changes in active and sedentary behaviors will also be used to determine the impacts of VR exergames on metabolic syndrome.

**Results:**

The ethics committee of Guangzhou University, China, approved this study on September 25, 2024. Participant recruitment will begin in early 2025 and continue for approximately 3 months. Data will be analyzed after the 12-week trial is completed, with full results expected to be presented in early 2026.

**Conclusions:**

This study explores an emerging topic by applying an at-home immersive VR exergame intervention, potentially contributing to understanding the effects of an exergame program on metabolic syndrome risk among office workers.

**Trial Registration:**

ClinicalTrials.gov NCT06556784; https://clinicaltrials.gov/study/NCT06556784

**International Registered Report Identifier (IRRID):**

PRR1-10.2196/64560

## Introduction

Noncommunicable diseases, such as cancer, cardiovascular diseases, diabetes, and heart disease, constitute the foremost cause of death internationally [1]. Noncommunicable diseases accounted for 63% or 36 million global deaths in 2008, which increased to 74% in 2019 [2,3]. The increasing prevalence of noncommunicable disease deaths worldwide has increased the need to identify modifiable risks in preventing and managing these diseases.

Metabolic syndrome is a common chronic disease characterized by the co-occurrence of several major biomarkers, including high-density lipoprotein cholesterol (HDL-C), low-density lipoprotein cholesterol (LDL-C), impaired fasting glucose, insulin, triglycerides, blood pressure, waist circumference, and BMI [4,5]. Moreover, as a route to many noncommunicable diseases, such as cancer, cardiovascular diseases, and type 2 diabetes [6-8], metabolic syndrome plays a pivotal role in health status. For example, several systematic reviews have shown that individuals with metabolic syndrome are approximately two to five times more susceptible to developing cardiovascular disease and type 2 diabetes [9-11]. Although metabolic syndrome is one of the threats to a healthy life [12], its prevalence is increasing globally [13]. For example, a study conducted in the United States reported an increase of more than 35% in the prevalence of metabolic syndrome among US adults from 1988-1994 to 2007-2012 [14]. A systematic review reported that approximately 20% of the adult population in most Asian Pacific countries is struggling with a metabolic syndrome epidemic [15]. However, as an effective approach to mitigate the risk and treatment of metabolic syndrome, physical activity is facing a worrisome status across all countries [16,17].

Employed adults, the largest population in the world, spend a large portion of their waking time in the workplace [18,19]. Especially in desk-based workplaces, most of this time is spent sitting [20-22]. For example, in desk-based workplaces, Japanese workers spend approximately 70% of their time sitting with a low level of physical activity [23]. With the ongoing advancement of technology, the worldwide phenomenon of physical inactivity among employed adults has been occurring increasingly in recent decades [24]. An example study conducted in China revealed that the energy expenditure of occupational activity decreased by more than 22% and that the energy expenditure of domestic activity decreased by more than 51% over 9 years [25]. Other studies in the United States reported a steadily increasing trend of low-activity occupations of 76% from 1950 to 2000 [26] and a decreasing occupation-related caloric expenditure of more than 124 calories per day from 1960 to 2008 [27]. Prolonged sitting time adversely affects cardiometabolic health among office workers, an already at-risk population for metabolic syndrome [28]. For example, a study conducted in Central Iran reported that approximately 36% of office workers had metabolic syndrome [29]. Other studies have revealed that approximately one in ten office workers are at risk of metabolic syndrome in Korea [30] and Great Britain [31]. Physical activity promotion interventions have a strong positive impact on people with metabolic syndrome [32]. Nevertheless, several factors, such as the nature of the job and lack of motivation and time [33-35] for exercise, remain key barriers for office workers to adhere to an active lifestyle. Consequently, gym attendance rates are low even among people paying for membership [36], and more than 75% of people fail to keep their health target for more than 3 years [37].

Exergames are defined as video games that require players to be physically engaged in them to play [38,39]. Several systematic and narrative reviews have provided preliminary evidence for the correlations between playing exergames and users’ physical activities [40,41]. For example, the contribution of exergames to increasing older adults’ health and wellness through physical activity has been validated [41]. Previous studies have demonstrated that exergames can be used as interventions to increase physical activity, particularly among obese individuals [42]. With the ongoing advancement of virtual reality (VR) technology, exergames have become more immersive for users. The concept of immersion is defined as “users’ engagement with a VR system that results in being in a flow state” [43]. Owing to the technology limitations of conventional VR, several shortages have been presented to users, such as difficulty in producing dynamic natural environments and interaction deficiencies in human-environment simulations. In contrast, with the development of visualizations and interactions with virtual environments, new immersive VR methods can address these limitations and lead to more accurate and efficient virtual environment assessments [44]. Additionally, its new application in exergame areas has already shown potential for promoting the health and well-being of some disabled youth [45]. Nevertheless, research on the health impact of immersive exergames remains rare. A search of the terms “immersive exergame” or “video game,” “physical activity” or “exercise,” “cardiometabolic” or “metabolic syndrome,” and “office worker” produced 0 results in the Scopus database as of May 6, 2024 [46]. Thus, using immersive exergames to promote physical activity in relation to cardiometabolic health is still in its infancy among office workers, with a need for increased knowledge about this topic. Therefore, as the first randomized controlled trial (RCT), to the best of our knowledge, the purpose of this study is to examine the effects of at-home immersive VR exergames on clinical risk factors among office workers, an already at-risk population for metabolic syndrome.

## Methods

### Study Design

RCTs, a top-level research methodology in the area of evidence-based medicine [47], have played a significant role in evaluating the effects of lifestyle changes on metabolic syndrome [48-50]. This protocol is a longitudinal pilot study that uses a 3-arm parallel-group RCT to examine the interventional efficacy of an at-home immersive VR exergame on several clinical risk factors of metabolic syndrome among office workers. On the basis of prior research on exercise and physical activity interventions [48,51], a 12-week duration was selected for this trial, as it is a commonly used period for evaluating the efficacy of interventions targeting metabolic syndrome. Several clinical risk factors for metabolic syndrome, as well as active and sedentary behavior, will be monitored in three stages during this 12-week trial. Stage 1 will be at the start of the trial, Stage 2 at week 6, and Stage 3 at the end of the trial. The therapeutic effects of the at-home immersive VR exergame intervention for metabolic syndrome will be evaluated by comparing these risk factors among three groups: the VR exergame intervention group, the regular physical activity control group, and the nonexercise control group. The study follows two checklists: the CONSORT-EHEALTH (Consolidated Standards of Reporting Trials Statement for Randomized Controlled Trials of Electronic and Mobile Health Applications and Online TeleHealth) [52] and the SPIRIT (Standard Protocol Items: Recommendations for Interventional Trials; Multimedia Appendices 1 and 2) [53,54].

### Participant Recruitment

For this exploratory study, a convenience sampling method will be used to recruit 120 office workers from six office-oriented information technology companies in Guangzhou City, Guangdong Province, located in Southern China. This group represents a high proportion of physically inactive individuals [55,56] and is at greater risk of developing metabolic syndrome [56-58]. Recruitment emails will be sent to participants via the Tencent Enterprise Mailbox [59], a platform that allows companies to create email accounts with their corporate domain. This platform serves as a communication and office tool to support enterprises in providing unified notifications to office workers and to support office workers in providing timely and efficient feedback. The inclusion criteria for participants are office workers who are 16-60 years old (based on the standard age of the Labor Law of the People’s Republic of China), capable of standing and exercising, and have no history of heart disease, cancer, or dementia. Additionally, participants should be interested in playing with the exergames and willing to adhere to a 12-week intervention exergame treatment because of the nature of the trial.

This research aims to examine the effects of at-home immersive VR exergames on the clinical risk factors for metabolic syndrome. Hence, individuals who are already engaged in more than 150 minutes per week of moderate to vigorous intensity physical activity will be excluded [60]. Additionally, pharmacological treatments, such as metabolic syndrome–related medicines (insulin sensitizers and biguanides, orlistat, sibutramine, etc), some surgeries (bariatric surgery), and traditional Chinese medicine (herbal medicine of ginseng, berberine, bitter melon, etc), are the other exclusion criteria because of their potential to influence cardiometabolic outcomes [61,62].

First, to encourage participation, some basic information related to exercise for health and immersive VR exergames will be introduced to potential participants. After a detailed explanation of what is needed during the trial, a precheck of the inclusion and exclusion criteria will be asked to identify eligible participants. Then, participants who meet the criteria will be instructed to review the information and provide informed consent and assent for this study. Given the nature of the clinical risk factors to be collected, a data collection center will be arranged at a cooperative clinic, and its address will be shared with all the participants for data collection and allocation of experimental equipment and material at the start of the trial.

### Intervention and Control Groups

**Figure 1:**
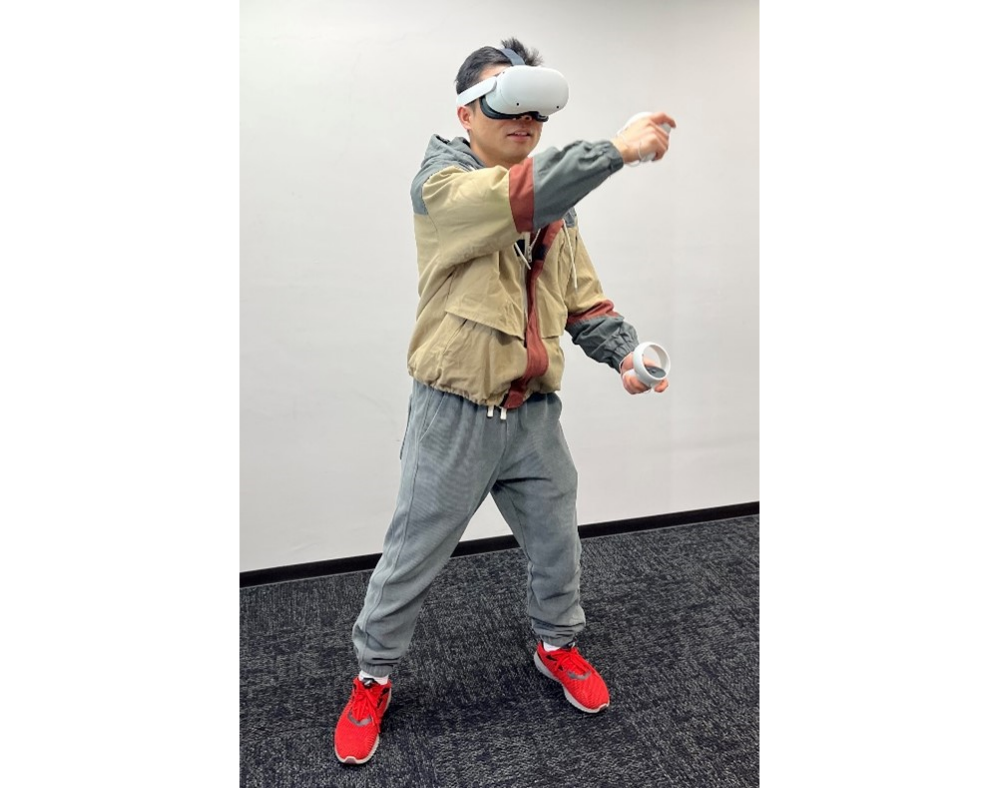
An example of exercising with aerobic immersive VR exergame using Meta Quest 2 (source: the authors). VR: virtual reality.

Following RCT guidelines [63,64], participants will be randomized into three equal groups: (1) the at-home immersive VR exergame intervention group, (2) the regular physical activity control group, and (3) the nonexercise control group. Including both the regular physical activity and nonexercise control groups will help determine whether VR exergame intervention offers unique benefits beyond those achieved through traditional exercise or no intervention. This design allows us to isolate the specific effects of immersive VR exergames. The at-home immersive VR exergame intervention includes a 12-week trial of exercise using Meta Quest 2, as shown in Figure 1. The Meta Quest 2 device is a valid tool that has been used in previous studies to support physical activity [65-68]. A VR headset and 2 handheld controllers are contained in the Meta Quest 2 set. Several active aerobic game apps will be installed on headset devices. These apps include the following [69]: Beat Saber (exercise: full-body, aerobic), Synth Riders (exercise: dance, aerobic), OhShape (exercise: dance, aerobic), Ragnarock (exercise: arms, shoulders, aerobic), Dance Central (exercise: dance, aerobic), Until You Fall (exercise: back, shoulders, arms, aerobic), In Death: Unchained (exercise: arms and shoulders, endurance/aerobic), and Space Pirate Trainer (exercise: full-body, aerobic). In these immersive games, the users can present their body movements in the game world from a first-person perspective. Moreover, they are all designed to motivate users to participate in physical movements with various categories of motivational affordances. For example, Beat Saber is a rhythm game in which users need to move their bodies to slice blocks at an exact angle. Physical activities such as arm waving, moving, and squatting with an accelerating speed are motivated in the game with affordances of rank, point, and record. The participants in the VR exergame intervention group will be instructed to play one or more of these exergames for at least 150 minutes per week, which is in line with the World Health Organization’s physical activity guidelines [60]. They will be requested to record the frequency and duration of their exergaming sessions. Participants in the regular physical activity control group will engage in a physical activity routine similar in duration and intensity to the VR exergame intervention. Participants in the nonexercise control group will not receive any specific exercise instructions. All groups will be advised to maintain regular eating and working habits without making significant changes throughout the 12-week trial. After completing the trial, participants in both control groups will be offered the same procedures as the intervention group, but their data will not be collected.

### Measures

This exploratory pilot study aims to test the intervention of at-home immersive VR exergame on metabolic syndrome. Two variables, including clinical risk factors for metabolic syndrome and active and sedentary behaviors, will be measured throughout the three stages of the trial. Demographic information will be collected only at stage 1. Major clinical risk factors are objectively measured from participants’ blood tests and a sphygmomanometer. The Global Physical Activity Questionnaire (GPAQ) will be used to measure participants’ active and sedentary behaviors. A summary of the stages, measures, and indicators is shown in Table 1.

**Table 1:** Summary of stages, measures, and indicators.

Measures and indicators		Timeline		
		Stage 1	Stage 2	Stage 3
**Demographics**				
	Age, gender	✓		
**Clinical risk factors**				
	HDL-C^a^	✓	✓	✓
	LDL-C^b^	✓	✓	✓
	Impaired fasting glucose	✓	✓	✓
	Insulin	✓	✓	✓
	Triglycerides	✓	✓	✓
	Blood pressure	✓	✓	✓
	Waist circumference	✓	✓	✓
	BMI	✓	✓	✓
**Active and sedentary behaviors**				
	Exercise time (including exergaming time)	✓	✓	✓
	Sedentary time	✓	✓	✓

^a^HDL-C: high-density lipoprotein cholesterol.

^b^LDL-C: low-density lipoprotein cholesterol.

To test the therapeutic effect of this intervention, primary clinical risk factors for metabolic syndrome, including blood tests for HDL-C, LDL-C, impaired fasting glucose, insulin, and triglycerides, along with sphygmomanometry of blood pressure, waist circumference, and BMI, will be measured [4,70,71]. Owing to several merits of immersive VR exergames, such as physical engagement [38,39], the nature of immersion, and attractiveness [43], the use of VR exergames may have a significant effect on behaviors, such as exercise and sitting time [72]. Therefore, we use the GPAQ to observe the changes in active and sedentary behaviors produced by immersive VR exergames. Moreover, the validity of the GPAQ has been proven for monitoring physical activity in health promotion research [73]. The whole questionnaire includes 16 items for 3 intensities (light, moderate, and vigorous intensity) of physical activity and sedentary behavior during work, transport, and leisure time [74]. The paper form will be printed and passed to participants to assess the changes in active and sedentary behaviors during the trial.

### Study Procedure

Each group in this study will consist of 40 randomly allocated participants (using a simple computer-generated random number sequence managed by JZ), and the trial will be conducted in a single-blinded structure. In this three-arm study, participants will receive the VR exergame intervention, regular physical activity, or no exercise at all. The participants will inherently be aware of their assigned activity. However, they will be blinded to the specific study hypotheses and the detailed objectives regarding comparing the 3 groups. To reduce perceived differences, participants in both control groups will receive VR exergame devices after completing the trial, but no data will be collected from this phase. Additionally, outcome assessors and data analysts will remain blinded to group assignments to prevent bias in data collection and analysis. This single-blind design helps reduce bias in the interpretation of the study results, even though participants are aware of their assigned activity.

The comprehensive framework for this protocol consists of 3 stages over 12 weeks and the allocation of items for each stage is shown in Table 2. In stage 1, when participants arrive at the data collection center, experiment coordinators will distribute and explain the manual booklet to all participants, which includes a description of the procedures, requirements, and address for inquiries. After participants have confirmed the contents and requirements of the manual, the experiment coordinators and clinic nurses will collect demographics, clinical risk factors, and GPAQ from all participants as the baseline data. A Meta Quest 2 set of equipment (entire trial), GPAQ questionnaire (0 to 6 weeks), and a recording sheet of exergaming time (0 to 6 weeks) will be additionally assigned to the VR exergame intervention group. In stage 2, the GPAQ questionnaire (7 to 12 weeks) will be assigned to all 3 groups and a recording sheet of exergaming time (7 to 12 weeks) will be additionally assigned to the VR exergame intervention group. In stage 3, only the Meta Quest 2 set of equipment will be assigned to both control groups. The necessary items for each stage will be passed to participants when they arrive at the data collection center.

**Table 2:** Timeline and allocation of items.

	Stage 1^a^	Stage 2^b^	Stage 3^c^
VR^d^ exergame intervention group	Manual booklet Demographic questionnaire GPAQ^e^ Meta Quest 2 set Recording sheet of exergaming time	GPAQ Recording sheet of exergaming time	—^f^
Regular physical activity control group	Manual booklet Demographic questionnaire GPAQ	GPAQ	Meta Quest 2 set
Nonexercise control group	Manual booklet Demographic questionnaire GPAQ	GPAQ	Meta Quest 2 set

^a^Start of the trial.

^b^Week 6 of the trial.

^c^End of the trial.

^d^VR: virtual reality.

^e^GPAQ: Global Physical Activity Questionnaire.

^f^Not applicable.

All participants will be asked to visit the data collection center in three stages for data collection and experimental material allocation. The self-reported GPAQ questionnaire will be completed by all participants at home, as it is a convenient at-home format for exploring impacts on active and sedentary behavior. The VR exergame intervention group will record the frequency and duration of their exergaming sessions, the regular physical activity control group will track their daily exercise routines, and the nonexercise control group will report any physical activities they perform beyond their baseline. Participants will be asked to bring all self-report sheets to the data collection center at stages 2 and 3. Coordinators will collect and summarize all the completed questionnaires and recording sheets for data processing. Major clinical risk factors are objectively measured from participants’ metabolic syndrome biomarkers, which are operated by professional nurses at the data collection center (cooperative clinic). Since there is a fasting biomarker for testing metabolic syndrome, all participants are required not to consume any food or drink 12 hours before blood sample collection in the morning. After the major clinical risk factor data from all the participants for the entire trial are collected, the experiment coordinators will visit and obtain the data from the data collection center.

### Analyses

After 12 weeks of the trial, 3 categories of data, including demographic, clinical risk factor, and active and sedentary behavior data, will have been collected. Regarding descriptive statistics for each indicator, the changes in clinical risk factors during the first 6 weeks, the last 6 weeks, and the entire 12-week trial will be calculated and presented as the means, SDs, and quartiles to show differences. Active and sedentary behavior will be presented as minutes of weekly light, moderate, and vigorous-intensity physical activity, and sedentary time will be obtained from the self-reported GPAQ items. Demographic variables will be presented as sum values or percentages. Additionally, the diagnosis of metabolic syndrome for all groups of participants will be summarized into sums and percentages at each stage according to the definition from a World Health Organization consultation report (high levels of insulin and impaired fasting glucose, together with at least two of the following items: HDL-C concentration less than 35 mg/dL for men [39 mg/dL for women], triglyceride concentration greater than 150 mg/dL, a waist circumference greater than 37 inches, BMI greater than 30 kg/m^2^, systolic blood pressure greater than 140 mm Hg and diastolic blood pressure greater than 90 mm Hg) [4,70].

As it will be adjusted for demographic variables, the linear mixed model, which is recommended in the literature for individually assigned RCTs [75], will be used as an analysis method for determining the effects of the intervention. In cases where participants drop out or are nonadherent, missing data will be handled via multiple imputation techniques. The primary outcome assessed is the change in clinical risk factors for metabolic syndrome, including HDL-C, LDL-C, triglycerides, blood pressure, waist circumference, BMI, impaired fasting glucose, and insulin. The linear mixed model will compare these clinical risk factors across the 3 groups—VR exergame intervention, regular physical activity control, and nonexercise control groups—over three stages. The model will also assess the interaction between groups and time to determine whether the VR exergame intervention has different or additional effects compared with regular physical activity and no physical activity. Adherence to the intervention will be assessed through daily self-report logs for all participants and included as a covariate to account for variations in engagement with the interventions. Additionally, sensitivity analyses will be conducted to examine the potential impact of varying adherence levels on metabolic outcomes. This approach ensures that adherence variations are properly considered when the study’s results are interpreted. In this study, the intervention or control groups are fixed factors, and demographic factors would be considered as random factors for accommodating variabilities within or between participants. All available data will be imported into Stata (version SE 15.1; Stata Corp) for analysis, and the significance level will be set at *P*<.05. As this is an exploratory pilot study, the primary objective is to assess feasibility and gather preliminary data. Given the lack of prior studies using immersive VR exergames in this context, conducting a power analysis would be speculative. The focus of this pilot is to generate effect size estimates and assess practical considerations, which will inform the design of a future, fully powered RCT. In the larger RCT, a formal power analysis will be conducted using the data generated from this pilot study.

### Ethical Considerations

This study was approved by the Institutional Ethics Committee of Guangzhou University, China (2024-112; September 25, 2024). Initially, potential participants will be provided with basic information regarding the benefits of exercise for health and immersive VR exergames. Subsequently, interested individuals will receive a detailed explanation of the study process, and they will be asked to sign the informed consent and assent forms. In addition, some personal information is required during this trial, including name, age, gender, email address, home address, and phone number for the purpose of marking data, sending the results of blood tests and diagnosis of metabolic syndrome, or analyses (eg, as covariates). All participant information will be collected directly by the experiment coordinator (JZ) to ensure the privacy of their personal information. Strict measures will be implemented to manage and store all the collected data. To compensate for participation, participants will be rewarded with RMB 150 (approximately US $21) for completing each stage, whereas participants who complete all the processes of the trial will be rewarded with RMB 500 (approximately US $71). All incentives will be sent via a registered postal cash envelope. For visiting the data collection center at each stage, transportation fees will also be reimbursed.

## Results

This study was approved by the ethics committee of Guangzhou University, China, as of September 25, 2024. The completion of participant recruitment and the initiation of the intervention or control trial are anticipated by the middle of 2025. The full processes of this trial, including data analysis and paper writing, are expected to be completed in early 2026.

## Discussion

### Principal Findings

Our findings will reveal whether the clinical risk factors for metabolic syndrome within the intervention group significantly improved after a 12-week exergaming intervention compared with those in the two control groups. The increase in the time engaged in physical activity (all types of light, moderate, and vigorous intensity) and the decrease in the time spent in sedentary behavior will also be evaluated.

The phenomenon of physical inactivity among employed adults has been occurring increasingly worldwide because of the development of society and technology in recent decades [16,17]. Consequently, the population of employed adults at risk for metabolic syndrome is growing across the globe [14,15]. Several primary factors, such as a lack of motivation, weather restrictions, and busy schedules for work, social lives, and family, have been reported as key barriers to physical activity [34]. Notably, at-home immersive VR exergames can provide several advantages to address these barriers to physical inactivity for employed adults. However, to our knowledge, few studies have explored the health effects of immersive exergames. This study is the first RCT and aims to examine the effectiveness of at-home immersive VR exergame interventions for improving metabolic syndrome. Our hypotheses were based on several advantages of at-home immersive VR exergames. The first benefit to exergames is engagement and enjoyment, as various types of motivational affordances (eg, points, leaderboard, and badges) were found within the gaming process along with several positive psychological effects (eg, joyfulness) [76]. The second advantage is the convenience of at-home exercise, which can mitigate weather restrictions and fit into a flexible schedule. In addition, exergames also have positive impacts on both physical health and cognition [77], which may offer pathways to improve rehabilitation and wellness [41]. Although these positive results have been reported, there is also some evidence that these positive impacts from exergames may be temporary [78].

### Strengths and Limitations

This study has several limitations. First, the possibility of VR sickness resulting from individual differences [79] may result in variable exercise time, which can indirectly influence the primary clinical risk factors for metabolic syndrome and bias the efficacy of the intervention. Second, the unsupervised process of self-recorded exercise time and self-reported GPAQ items may produce inexact data about trial results. Third, according to trial length within exercise or physical activity studies, 12 weeks to 1 year is the range for determining interventional efficacy for comprehensive metabolic syndrome risk factors [48,51]. The 12-week trial for this study is consistent with the minimum length threshold, but a longer treatment period may produce more robust and efficacious results. Additionally, our participants will be recruited from Guangzhou, China, where particular life habits and social conventions, such as long work hours [80] and food habits [81], may produce results with limited generalizability. Although these limitations exist, the strengths of this study include its RCT design, the use of clinical measures of risk factors for metabolic syndrome, the novel examination of the impacts of exergames on promoting physical activity and metabolic syndrome, and the improvement of health conditions within the at-risk population of office workers.

### Conclusions

The use of at-home immersive VR exergames for improving metabolic syndrome is an emerging topic for promoting physical activity related to cardiometabolic health. The primary aim of this proposed study is to assess the feasibility and preliminary effects of an at-home immersive VR exergame intervention on the clinical risk factors for metabolic syndrome. The results of using at-home immersive VR exergames may contribute to promoting office workers’ health conditions and help reduce the burden of metabolic syndrome risk among a large and increasingly sedentary population. These results will inform the design and implementation of a larger-scale, fully powered RCT. This exploratory pilot RCT will provide data regarding the intervention’s practicality and initial effectiveness, which will guide future research efforts.

## Appendix

Multimedia Appendix 1 CONSORT-eHEALTH checklist (V 1.6.1).

Multimedia Appendix 2 SPIRIT (Standard Protocol Items: Recommendations for Interventional Trials) checklist.

## Abbreviations

CONSORT-EHEALTH: Consolidated Standards of Reporting Trials Statement for Randomized Controlled Trials of Electronic and Mobile Health Applications and Online TeleHealth
GPAQ: Global Physical Activity Questionnaire
HDL-C: high-density lipoprotein cholesterol
LDL-C: low-density lipoprotein cholesterol
RCT: randomized controlled trial
SPIRIT: Standard Protocol Items: Recommendations for Interventional Trials
VR: virtual reality
